# Plasticity of resting state brain networks in recovery from stress

**DOI:** 10.3389/fnhum.2013.00919

**Published:** 2013-12-27

**Authors:** José M. Soares, Adriana Sampaio, Paulo Marques, Luís M. Ferreira, Nadine C. Santos, Fernanda Marques, Joana A. Palha, João J. Cerqueira, Nuno Sousa

**Affiliations:** ^1^Life and Health Sciences Research Institute (ICVS), School of Health Sciences, University of MinhoBraga, Portugal; ^2^ICVS/3B's - PT Government Associate LaboratoryBraga/Guimarães, Portugal; ^3^Clinical Academic CenterBraga, Portugal; ^4^Neuropsychophysiology Lab, CIPsi, School of Psychology, University of MinhoBraga, Portugal

**Keywords:** resting state networks, functional connectivity, deactivation, recovery from stress, plasticity

## Abstract

Chronic stress has been widely reported to have deleterious impact in multiple biological systems. Specifically, structural and functional remodeling of several brain regions following prolonged stress exposure have been described; importantly, some of these changes are eventually reversible. Recently, we showed the impact of stress on resting state networks (RSNs), but nothing is known about the plasticity of RSNs after recovery from stress. Herein, we examined the “plasticity” of RSNs, both at functional and structural levels, by comparing the same individuals before and after recovery from the exposure to chronic stress; results were also contrasted with a control group. Here we show that the stressed individuals after recovery displayed a decreased resting functional connectivity in the default mode network (DMN), ventral attention network (VAN), and sensorimotor network (SMN) when compared to themselves immediately after stress; however, this functional plastic recovery was only partial as when compared with the control group, as there were still areas of increased connectivity in dorsal attention network (DAN), SMN and primary visual network (VN) in participants recovered from stress. Data also shows that participants after recovery from stress displayed increased deactivations in DMN, SMN, and auditory network (AN), to levels similar to those of controls, showing a normalization of the deactivation pattern in RSNs after recovery from stress. In contrast, structural changes (volumetry) of the brain areas involving these networks are absent after the recovery period. These results reveal plastic phenomena in specific RSNs and a functional remodeling of the activation-deactivation pattern following recovery from chronic-stress, which is not accompanied by significant structural plasticity.

## Introduction

When the homeostatic mechanisms are disrupted, namely through prolonged stress exposure, maladaptive responses take place and trigger inappropriate functional responses. It is well-established that prolonged stress has deleterious impact in multiple biological systems, including the central nervous system. In fact, prolonged stress exposure impairs spatial working memory, perceptual attention, behavioral flexibility, and decision making both in rodents and in humans (Joels et al., 2004; Cerqueira et al., 2005; Dias-Ferreira et al., 2009; Soares et al., 2012; Yuen et al., 2012), which has been associated with structural and functional changes of several brain regions. Importantly, some of these maladaptive structural and functional responses to increased chronic stress were shown to be reversible (Sousa et al., 1998; Heine et al., 2004; Cerqueira et al., 2005; Goldwater et al., 2009; Bian et al., 2012; Soares et al., 2012), including evidence showing that as trait positive affect may potentiate recovery and adaptive response (Papousek et al., 2010). However, certain stress effects and specific structural and functional changes may endure after this recovery period (Joels et al., 2004; Gourley et al., 2013). Of notice, most stress recovery studies were performed in rodent models.

A growing field of functional magnetic resonance imaging (fMRI) has provided new insights into the functional connectivity across different brain regions. Indeed, resting state fMRI is being widely used to assess brain regional interactions that comprise the resting state networks (RSNs) (De Luca et al., 2006; Fox and Raichle, 2007), both during resting periods and task-induced deactivations. Moreover, alterations in the normal patterns of RSNs have been associated with several disease states and neuropsychiatric disorders (Zhang and Raichle, 2010; Meda et al., 2012; Sripada et al., 2012), including stress exposure (Soares et al., 2013). Indeed, we previously reported that stressed participants had an hyperactivation pattern of the default mode (DMN), dorsal attention (DAN), ventral attention (VAN), sensorimotor (SMN), and primary visual (VN) networks, paralleled by structural constriction of the DMN brain regions (Soares et al., 2013).

The existence of plastic events in the RSNs after recovery from chronic stress is, however, largely unknown. Indeed, Vaisvaser et al. (2013), using an acute social stress model, examined stress-induced responses in the RSNs and cortisol levels before stress, immediately after the acute stress exposure and 2 h later. The authors found a “recovery” pattern of the DMN connectivity after stress exposure in two of the central hubs of the DMN (seed ROIs at the posterior cingulate cortex and hippocampus), but not in the amygdala-hippocampal disconnectivity that was sustained at 2 h post-stress. Moreover, this increased connectivity was inversely correlated with cortisol levels (Vaisvaser et al., 2013). These results suggest that even acute psychosocial stressors are associated with a prolonged post-stress DMN connectivity response in specific brain regions. This study used only an acute stress model and studies addressing how RSNs respond to chronic stress and identifying specific networks that are associated to an efficient recovery are absent. Therefore, the present study examined the effects of chronic stress on the RSNs following recovery and investigated region-specific changes during successful recovery from chronic stress exposure.

## Materials and methods

### Participants, psychological tests, and cortisol measurements

The participants included in this study were 6 stress participants submitted to prolonged psychological stress exposure (3 males, 3 females; mean age, 23.83 ± 0.37), the same 6 stress participants, 6 weeks after the end of the exposure to stress and 6 controls (3 males, 3 females; mean age, 24.33 ± 1.24). Control participants included a cohort of medical students under their normal academic activities, whereas the stress group included participants that had just finished their long period of preparation for the medical residence selection exam (stress group). Participants responded to a laterality test and to a self-administered questionnaire regarding stress assessment (Perceived Stress Scale—PSS) (Cohen et al., 1983). Participants were further assessed with the Hamilton anxiety scale—HAS (Hamilton, 1959) and the Hamilton depression scale—HDS (Hamilton, 1967) by a certified psychologist. Upon filling of the questionnaires, and immediately before the imaging acquisitions, participants collected saliva samples with the help of Salivette (Sarstedt, Germany) collection devices. Collection took place between 9 and 5 p.m. in all participants. Samples were stored at −20°C until the biologically active, free fraction of the stress hormone cortisol was analyzed using an immunoassay (IBL, Hamburg).

### Ethics statement

The study was conducted in accordance with the principles expressed in the Declaration of Helsinki and was approved by the Ethics Committee of Hospital de Braga (Portugal). The study goals and tests were explained to all participants and all gave informed written consent.

### Data acquisition

Participants were scanned on a clinical approved Siemens Magnetom Avanto 1.5 T (Siemens Medical Solutions, Erlangen, Germany) on Hospital de Braga using the Siemens 12-channel receive-only head coil. The imaging sessions, including one structural T1, one resting state functional, and two task related functional acquisitions, were conducted in the same day and the Siemens Auto Align scout protocol was used to minimize variations in head positioning. For structural analysis, a T1 high-resolution anatomical sequence, 3D MPRAGE (magnetization prepared rapid gradient echo) was performed with the following scan parameters: repetition time (*TR*) = 2.4 s, echo time (*TE*) = 3.62 ms, 160 sagittal slices with no gap, field-of-view (*FoV*) = 234 mm, flip angle (*FA*) = 8°, in-plane resolution = 1.2 × 1.2 mm^2^ and slice thickness = 1.2 mm. During resting-state fMRI acquisition, using gradient echo T2^*^ weighted echo-planar images (EPIs), participants were instructed to keep the eyes closed and to think about nothing in particular. The imaging parameters were: 100 volumes, *TR* = 3 s, *TE* = 50 ms, *FA* = 90°, in-plane resolution = 3.4 × 3.4 mm^2^, 30 interleaved slices, slice thickness = 5 mm, imaging matrix 64 × 64 and *FoV* = 220 mm. fMRI paradigm acquisition was acquired using: *TR* = 2 s, *TE* = 20 ms, *FA* = 90°, in-plane resolution and slice thickness 3.3 mm, 38 ascending interleaved axial slices with no gap and *FoV* = 212 mm. The functional paradigm acquisitions were previously described (Soares et al., 2012) and the paradigm was presented using the fully integrated fMRI system IFIS-SA.

### Image pre-processing

Before any data processing and analysis, all acquisitions were visually inspected and confirmed that they were not affected by critical head motion and that participants had no brain lesions.

To achieve signal stabilization and allow participants to adjust to the scanner noise, the first 5 resting state fMRI volumes (15 s) were discarded. Data preprocessing was performed using SPM8 (Statistical Parametrical Mapping, version 8, http://www.fil.ion.ucl.ac.uk) analysis software. Images were firstly corrected for slice timing using first slice as reference and SPM8's Fourier phase shift interpolation. To correct for head motion, images were realigned to the mean image with a six-parameter rigid-body spatial transformation and estimation was performed at 0.9 quality, 4 mm separation, 5 mm FWHM smoothing kernel using 2nd degree B-Spline interpolation. No participants exceed head motion higher than 2 mm in translation or 1° in rotation. Images were then spatially normalized to the MNI (Montreal Neurological Institute) standard coordinate system using SPM8 EPI template and trilinear interpolation. Data were then re-sampled to 3 × 3 × 3 mm3 using sinc interpolation, smoothed to decrease spatial noise with a 8 mm, full-width at half-maximum (FWHM), Gaussian kernel, temporally band-pass filtered (0.01–0.08 Hz) and the linear trend was removed. The pre-processing of fMRI paradigm images was previously described (Soares et al., 2012).

### Independent component analysis and identification of RSN

Spatial independent component analysis was conducted for using the Group ICA 2.0d of fMRI Toolbox (GIFT, http://www.icatb.sourceforge.net) (Calhoun et al., 2001; Correa et al., 2005). Concisely, spatial ICA analysis is a fully data-driven approach that consists in extracting the non-overlapping spatial maps with temporally coherent time courses that maximize independence. The methodology employed by GIFT can be summarized in three main stages: dimensionality reduction, estimation of the group independent components, and back-reconstruction of each subject's corresponding independent components. The reduction of dimensionality of the functional data and computational load was performed with Principal Component Analysis (PCA) in the concatenated dataset over all subjects, independently of the groups. Then, 20 independent components were estimated, based on a good trade-off (clustering/splitting) between preserving the information in the data while reducing its size (Beckmann et al., 2005; Zuo et al., 2010), using the iterative Infomax algorithm. The ICASSO tool was used to assess the ICA reliability, and 20 computational runs were performed on the dataset, during which the components were being recomputed and compared across runs and the robustness of the results was ensured (Himberg et al., 2004). The previous steps result in the estimation of a mixing matrix with partitions, unique to each subject. The individual independent components were then back-reconstructed from the group-level components. This back-reconstruction step is accomplished by projecting each subject's data onto the inverse of the partition of the calculated matrix corresponding to that subject. The obtained independent components were expressed in t-statistic maps, which were finally converted to a z-statistic. Z-statistic describes the voxels that contributed more intensely to a specific independent component, providing a degree of functional connectivity within the network (Bartels and Zeki, 2005; Beckmann et al., 2005). The final components were visually inspected, sorted, and spatially correlated with resting state functional networks from (Shirer et al., 2012). Each subject's map corresponding to the best-fit component of each RSN was used to perform group statistical analyses.

### RSN deactivation during fMRI task analysis

The fMRI decision-making paradigm analyzed to investigate the task-induced deactivations consisted of two different event-related jittered design sessions. First session of valued actions with reward delivery and, after 30 min break, the second session consisted of the devalued actions with the outcome devaluation and extinction. Both sessions had 150 trials, each with 1.5 s for decision, 4 s with the choice highlighted, and 2 s for reward delivery, followed by the interstimulus interval with mean duration of 4 s [please see Soares et al. (2012), for further details].

fMRI paradigm was analyzed by creating a set of regressors at resting and decision making periods, which were convolved with the hemodynamic response function. In order to reliably map task-induced deactivations, we combined all the resting periods (resting baseline condition) and all the decision periods (decision condition), given that decision periods were equally demanding. The contrast used to assess task-induced deactivations was the resting baseline condition minus decision condition. Resulting functional patterns were masked with the previously described RSNs masks (Shirer et al., 2012).

### Structural analysis

Structural analysis based on segmentation of brain structures from T1 high-resolution anatomical data was performed using the freely available Freesurfer toolkit version 5.0 (http://surfer.nmr.mgh.harvard.edu). Intracranial volume (ICV) was used to correct the volumes and the processing pipeline was the same as previously described (Soares et al., 2012). DMN was defined by the summed volume of the angular gyrus of inferior parietal lobe, the posterior cingulate, the precuneus, and the frontopolar region (Raichle et al., 2001; Buckner et al., 2008). The summed volume of the middle frontal gyrus (dorsolateral and prefrontal region) and the posterior parietal region constituted the DAN (Seeley et al., 2007; Sridharan et al., 2008). VAN was constituted by the sum of the temporal-parietal junction and the ventral frontal cortex volumes (Fox et al., 2006). SMN was defined by the summed volume of the paracentral, precentral postcentral, and the cerebellum (Shirer et al., 2012). The summed volume of the cuneus, pericalcarine, and the lingual region constituted the primary VN (Shirer et al., 2012). Auditory network (AN) was defined by the summed volume of the temporal transverse and the temporal superior (Shirer et al., 2012).

### Statistical analyses

Results of the psychological scales, cortisol levels, and regional volumes were analyzed in the IBM SPSS Statistics software, v.19 (IBM, New York). Comparisons between the stress recovered and stress were done with paired samples *t*-test and between stress recovered and control with two-tailed independent-samples *t*-test. For all these comparisons significance level was set at 0.05. Values are presented as mean ± standard error of the mean.

Group analysis of the resting state fMRI and task induced deactivations were performed using the second level random effect analyses in SPM8. Initially, within group analyses were performed only to confirm the functional connectivity of the RSNs in the different groups, using one-sample *t*-tests. Therefore, between group analyses were implemented with directional two-sample *t*-tests. Functional results for all RSNs were considered significant at *p* < 0.05 corrected for multiple comparisons using a combination of an uncorrected height threshold of *p* < 0.025 with a minimum cluster size. The cluster size was determined over 1000 Monte Carlo simulations using AlphaSim program distributed with REST software tool (http://resting-fmri.sourceforge.net/). AlphaSim input parameters were the following: individual voxel probability threshold = 0.025, cluster connection radius = 3 mm, gaussian filter width (FWHM) = 8 mm, number of Monte Carlo simulations = 1000 and mask was set to the corresponding RSN template mask. Anatomical labeling was defined by a combination of visual inspection and Anatomical Automatic Labeling atlas (AAL) (Tzourio-Mazoyer et al., 2002).

## Results

### Physiological and behavioral results

**Figure 1:**
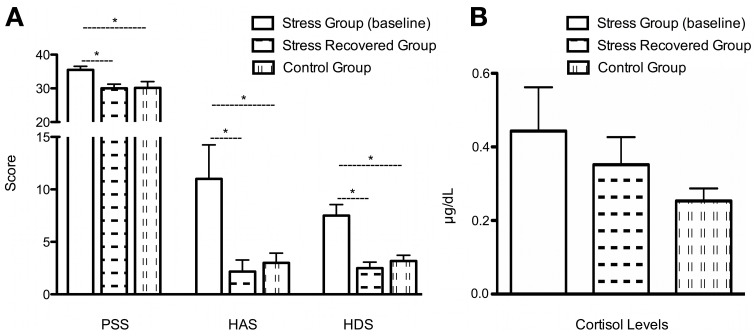
**Clinical characteristics of the cohort. (A)** Perceived Stress Scale (PSS), Hamilton Anxiety Scale (HAS); Hamilton Depression Scale (HAD) and **(B)** Salivary Cortisol levels in the two groups (stress, stress recovered, and control group); ^*^P < 0.05.

Stress impact was confirmed in several parameters: PSS [Figure 1A; *t*_(10)_ = 2.52, *P* < 0.05; Stress Group *M* = 35.50; *SD* = 2.59; Control Group *M* = 30.17; *SD* = 4.49] and in the HAS [Figure 1A; *t*_(10)_ = 2.37, *P* < 0.05 Stress Group *M* = 11.00; *SD* = 7.95; Control Group *M* = 3.00; *SD* = 2.28], and depression scores [HAD, Figure 1A; *t*_(10)_ = 3.65, *P* < 0.01; Stress Group *M* = 7.50; *SD* = 2.59; Control Group *M* = 3.17; *SD* = 1.33], but only by a trend when it regards to salivary cortisol levels [Figure 1B; *t*_(10)_ = 1.69, *P* = 0.12; Stress Group *M* = 0.44; *SD* = 0.29; Control Group *M* = 0.23; *SD* = 0.10]. After a stress-free period of 6 weeks after the end of the stress exposure, we observed that all the psychological changes were restored [Figure 1A; SPP: *t*_(5)_ = 3.72, *P* < 0.05; Stress Group *M* = 35.50; *SD* = 2.59; Stress-Recovered Group *M* = 30.00; *SD* = 3.03; anxiety score: *t*_(5)_ = 2.86, *P* < 0.05, Stress Group *M* = 11.00; *SD* = 7.95; Stress-Recovered Group *M* = 2.17; *SD* = 2.71]; depression score: HAD: [*t*_(4)_ = 4.84, *P* < 0.01; Stress Group *M* = 7.50; *SD* = 2.59; Stress-Recovered Group *M* = 2.5; *SD* = 0.35], except salivary cortisol levels [*t*_(5)_ = 0.67, *P* = 0.53; Stress Group *M* = 0.44; *SD* = 0.29; Stress-Recovered Group *M* = 1.38; *SD* = 0.8]. Importantly, stress-recovered group did not differ with the control group in all psychological and salivary cortisol measures [PSS: *t*_(10)_ = −0.08, *P* = 0.94; HAS: *t*_(10)_ = −0.58, *P* = 0.58; HAD: *t*_(10)_ = −0.85, *P* = 0.41; (Figure 1B) Cortisol: *t*_(10)_ = 1.42, *P* = 0.19].

### Functional connectivity results

The ICA analysis revealed the typical spatial pattern of functional connectivity and deactivation in DMN, DAN, VAN, SMN, VN, and AN in all experimental conditions (results not shown).

#### RSNs in stress and stress—recovered groups

**Figure 2:**
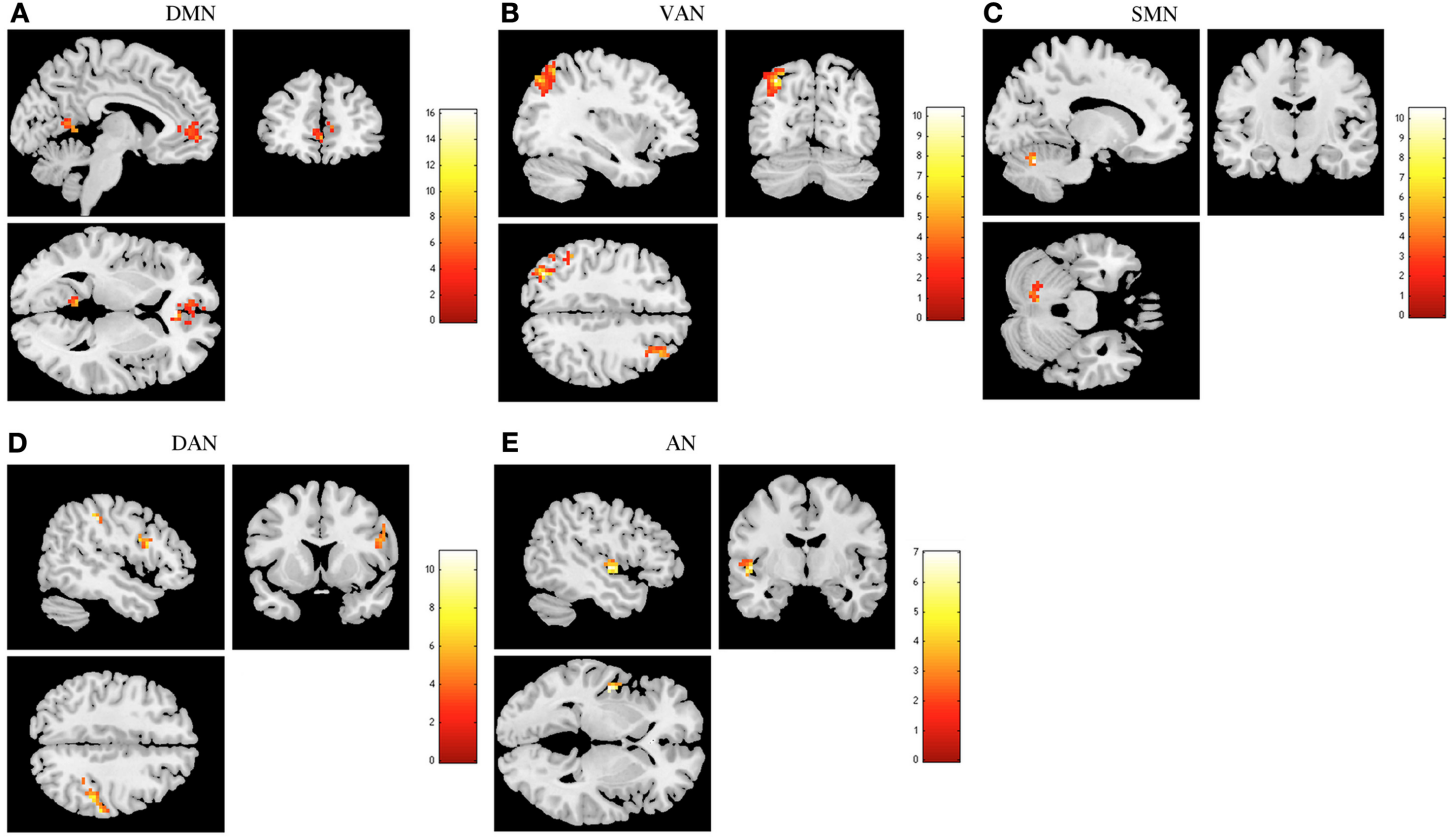
**The recovery from stress in resting state networks (RSNs) at rest**. The images depict areas in which stress participants display greater functional connectivity than stress recovered in the default mode network (DMN) **(A)**, ventral attention network (VAN) **(B)** and sensorimotor network (SMN) **(C)** and lower functional connectivity in the dorsal attention network (DAN) **(D)** and auditory network (AN) **(E)**. Results were extracted by independent component analysis and using paired *t*-tests, with results considered significant at a corrected for multiple comparisons *p* < 0.05 threshold.

Increased resting functional connectivity was identified in DMN, VAN, and SMN and decreased connectivity in DAN and AN in the stress group when compared to stress recovered participants (Figure 2 and Table 1).

**Table 1:** **Group differences (Stress vs. Stress recovered) at rest, in brain regions of the DMN, VAN, SMN, DAN, and AN maps (paired *t*-tests, corrected for multiple comparisons, *p* < 0.05)**.

	**Condition**	**Regions**	**Peak MNI coordinates**	**Cluster size (voxels)**	**Maximum *Z* score**
Stress > Stress recovered	Default mode network	Cingulum anterior (left)	0, 36, 3	141	4.31
		Frontal medial orbitofrontal (left)	−6, 54, −3		3.32
		Precuneus (right)	9, −63, 27	137	4.17
		Lingual (left)	−9, −48, 3		3.60
	Ventral attention network	Parietal inferior (left)	−33, −69, 45	225	3.80
		Parietal superior (left)	−33, −60, 48		3.64
		Frontal middle (right)	33, 36, 39	108	3.43
		Frontal superior (right)	30, 9, 63		2.97
	Sensorimotor network	Cerebellum (left)	−15, −63, −24	49	3.82
Stress < Stress recovered	Dorsal attention network	Parietal inferior (right)	45, −36, 48	81	3.87
		Supramarginal (right)	51, −30, 42		3.84
		Frontal inferior opercularis (right)	51, 15, 33	58	3.45
		Precentral (right)	51, 6, 27		3.22
	Auditory network	Temporal superior (left)	−48, −9, 0	52	3.32

Regarding DMN, stress group displayed increased functional connectivity mainly in the left cingulum, frontal medial orbitofrontal, right precuneus, and in the left lingual (Table 1). Increased functional connectivity was also found in VAN in stress group in the left parietal inferior and superior, right middle and superior frontal regions (Table 1) whereas in the SMN, increased functional connectivity was found in the left cerebellum (Table 1). In contrast, decreased functional connectivity was found in stress group in DAN, namely in the right parietal inferior, supramarginal, frontal inferior opercularis, and precentral regions (Table 1) as well as in the AN (left superior temporal region) (Table 1).

#### RSNs in stress—recovered and control groups

**Figure 3:**
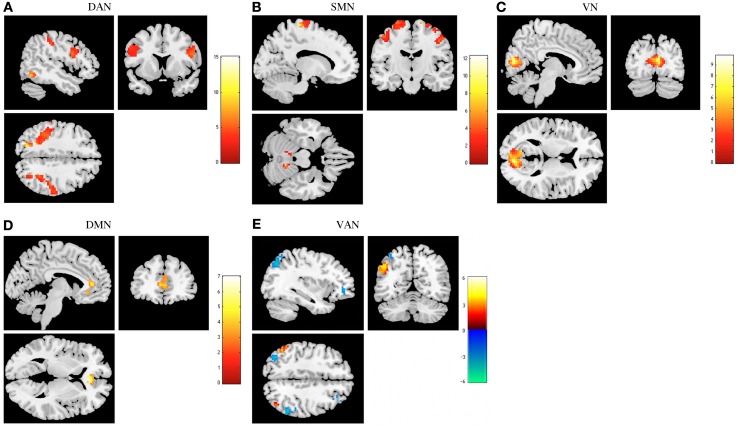
**Comparison between stress recovered participants and controls in resting state networks (RSNs)**. The images show areas in which stress recovered participants display greater functional connectivity than controls in the dorsal attention network (DAN) **(A)**, sensorimotor network (SMN) **(B)**, and primary visual network (VN) **(C)**. Lower functional connectivity was found in the default mode network (DMN) **(D)**. Ventral attention network (VAN) **(E)** displays increased functional connectivity in different regions both in stress recovered (orange) and in control (blue) participants. Results were extracted by independent component analysis and using two-sample *t*-tests, with results considered significant at a corrected for multiple comparisons *p* < 0.05 threshold.

Regarding the functional connectivity comparison between stress-recovered and controls, we found that the former presented an increased functional connectivity in the DAN, SMN, and VN. Increased connectivity in the left superior occipital, bilateral superior parietal, right postcentral, left middle and superior frontal, bilateral inferior frontal opercularis and bilateral precentral was found in the DAN of stress-recovered compared to control group (Table 2). A differential pattern of functional connectivity was observed for the VAN that is, while the stress-recovered group presented higher connectivity in the left inferior parietal and bilateral angular, they presented decreased functional connectivity in the bilateral inferior parietal, left angular, bilateral middle frontal and left inferior frontal triangularis. Additionally, stress-recovered group showed decreased connectivity in the DMN in the right anterior cingulate, in the SMN in the bilateral precentral, left paracentral, right postcentral, and bilateral cerebellum and in the VN in the bilateral calcarine (Table 2) when compared to controls (Figure 3).

**Table 2:** **Group differences (Stress recovered vs. Controls) at rest, in brain regions of the DAN, VAN, SMN, VN, and DMNN maps (two sample *t*-tests, corrected for multiple comparisons, *p* < 0.05)**.

	**Condition**	**Regions**	**Peak MNI coordinates**	**Cluster size (voxels)**	**Maximum *Z* score**
Stress recovered > Controls	Dorsal attention network	Occipital superior (left)	−24, −75, 42	504	5.53
		Parietal superior (left)	−24, −66, 54		5.21
		Parietal superior (right)	21, −66, 57	322	5.05
		Postcentral (right)	57, −21, 48		4.54
		Frontal superior (left)	−27, −6, 63	65	4.15
		Frontal middle (left)	−30, −3, 54		3.96
		Frontal inferior opercularis (right)	55, 15, 33	93	3.93
		Precentral (right)	51, 6, 24		3.85
		Precentral (left)	−51, 6, 27	92	3.15
		Frontal inferior opercularis (left)	−39, 3, 27		2.74
	Ventral attention network	Parietal inferior (left)	−54, −48, 39	136	3.35
		Angular (left)	−48, −66, 33		3.14
		Angular (right)	45, −60, 36	57	2.73
	Sensorimotor network	Precentral (left)	−27, −21, 78	333	5.17
		Paracentral (left)	−15, −27, 72		4.46
		Precentral (right)	15, −18, 75	237	4.33
		Postcentral (right)	30, −30, 60		3.80
		Cerebellum (right)	12, −51, 21	72	3.77
		Cerebellum (left)	−9, −48, −15		2.74
	Visual network	Calcarine (right)	6, −78, 12	331	4.77
		Calcarine (left)	−12, −66, 12		3.93
Stress recovered < Controls	Default mode network	Cingulum anterior (right)	6, 36, 18	147	3.47
	Ventral attention network	Parietal inferior (left)	−33, −74, 51	101	4.21
		Angular (left)	−36, −69, 45		2.78
		Frontal middle (right)	33, 27, 39	75	3.77
		Frontal inferior triangularis (left)	−48, 42, 0	59	3.08
		Frontal middle (left)	−36, 45, 0		2.64
		Parietal inferior (right)	51, −48, 48	58	3.04

#### Task-induced deactivations in stress and stress—recovered groups

**Figure 4:**
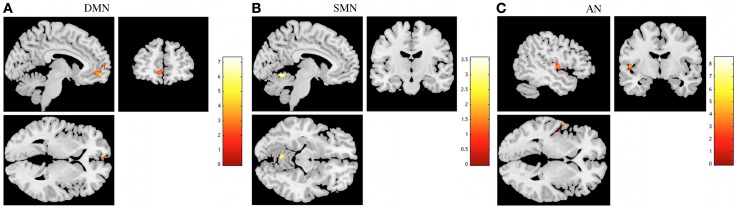
**The recovery from stress in resting state networks (RSNs) during task-induced deactivations**. The images illustrate areas of decreased deactivation in stress group when compared to stressed recovered participants in the default mode network (DMN) **(A)**, sensorimotor network (SMN) **(B)**, and auditory network (AN) **(C)**, extracted by general linear model analysis and using paired *t*-tests, with results considered significant at a corrected for multiple comparisons *p* < 0.05 threshold. Importantly, no areas of increased deactivation of these RSNs were found in stressed individuals when compared to stress recovered.

In task-induced deactivations, decreased deactivations in DMN, SMN, and AN were found in stress group when compared to stress-recovered participants (Figure 4 and Table 3). More specifically, decreased deactivations in the left medial frontal orbitofrontal and superior medial frontal were found in DMN of stress group (Table 3). In SMN, stress group presented lower functional deactivation in the left cerebellum (Table 3). The left superior temporal and rolandic operculum in AN were less deactivated in stress group compared to stress-recovered participants (Table 3). No significant region was found to display greater deactivation in stressed participants than in stress recovered in any of the studied RSNs.

**Table 3:** **Group differences (Stress < Stress recovered) in brain regions of the DMN, SMN, and AD maps in task-induced deactivation (paired *t*-tests, corrected for multiple comparisons, *p* < 0.05)**.

	**Condition**	**Regions**	**Peak MNI coordinates**	**Cluster size (voxels)**	**Maximum *Z* score**
Stress < Stress recovered	Default mode network	Frontal medial orbitofrontal (left)	−6, 56, −8	173	3.11
		Frontal superior medial (left)	−4, 64, 6		2.86
	Sensorimotor network	Cerebellum (left)	−4, −58, −8	57	2.27
	Auditory network	Temporal superior (left)	−58, 0, 2	82	3.28
		Rolandic operculum (left)	−50, −6, 4		2.48

#### Task-induced deactivations in stress—recovered and control groups

**Figure 5:**
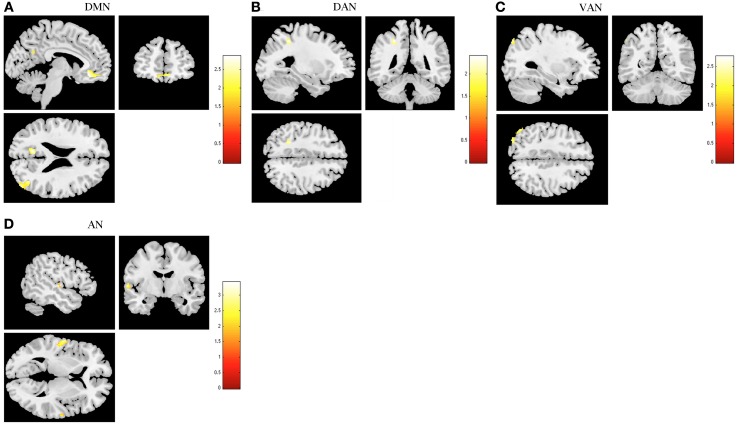
**Comparison between stress recovered participants and controls in resting state networks (RSNs) during task-induced deactivations**. The images demonstrate areas of decreased deactivation in stress-recovered participants when compared to controls in the default mode network (DMN) **(A)**, dorsal attention network (DAN) **(B)**, ventral attention network (VAN) **(C)**, and auditory network (AN) **(D)**, extracted by general linear model analysis and using two-sample *t*-tests, with results considered significant at a corrected for multiple comparisons *p* < 0.05 threshold. Importantly, no areas of increased deactivation of these RSNs were found in stress recovered when compared to controls participants.

To test for the degree of plasticity in RSNs, we compared deactivation between stress-recovered participants and controls. In this comparison, we found decreased deactivations in DMN, both attention networks, and AN (Figure 5 and Table 4) in stress-recovered group. In DMN, stress-recovered group showed decreased deactivations in the left cuneus, anterior cingulate, right medial frontal orbitofrontal, fusiform and middle temporal and in the left inferior parietal in DAN (Table 4). In VAN, stress-recovered group showed lower deactivation in the left superior parietal and in AN in the bilateral superior temporal (Table 4). No significant region was found to display greater deactivation in stress recovered than in control participants in any of the studied RSNs.

**Table 4:** **Group differences (Stress recovered < Controls) in brain regions of the DMN, DAN, VAN, and AN maps in task-induced deactivation (two sample *t*-tests, corrected for multiple comparisons, *p* < 0.05)**.

	**Condition**	**Regions**	**Peak MNI coordinates**	**Cluster size (voxels)**	**Maximum *Z* score**
Stress recovered < Controls	Default mode network	Cuneus (left)	−12, −58, 24	74	2.58
		Cingulum anterior (left)	−4, 34, −8	260	2.56
		Frontal medial orbitofrontal (right)	6, 52, −12		2.29
		Fusiform (right)	26, −36, −16	52	2.46
		Temporal middle (right)	42, −66, 22	157	2.44
	Dorsal attention network	Parietal inferior (left)	−26, −44, 48	41	2.23
	Ventral attention network	Parietal superior (left)	−28, −80, 50	57	2.52
	Auditory network	Temporal superior (left)	−62, −8, 6	129	3.00
		Temporal superior (right)	64, −12, 6	31	2.65

### Expansion/contraction maps of the RSNs

Whole brain analysis for relative ICVs did not differ between experimental groups. We showed in a previous study (Soares et al., 2013) that exposure to stress triggered a significant reduction in total DMN volume (corrected for ICV) with specific contraction in the left pCC, and bilateral parietal inferior brain regions. Herein, however, we did not find any significant differences in the volume of any of the RSNs between stress participants before and after recovery from stress. No significant areas of expansion or constriction were found in the dorsal and ventral attention networks, SMN, AN, and primary VN between stress and stress-recovered participants (*p* = 0.99, *p* = 0.98, *p* = 0.87, *p* = 0.84, and *p* = 0.99, respectively) and between stress-recovered and control groups (*p* = 0.89, *p* = 0.54, *p* = 0.18, *p* = 0.47, and *p* = 0.87, respectively).

## Discussion

In this study, we analzsed how the RSNs respond and change following recovery after chronic stress exposure. Our hypothesis was of a continuous recovery effect, in which the connectivity would be decreasing from stress toward the control group. Indeed, we observed a decreased resting functional connectivity in the DMN, VAN, and SMN after stress recovery. Additionally, decreased functional connectivity was also observed in the DAN, SMN, and VN networks in controls, when compared with stress-recovered group. However, only a specific brain region of the DMN (the right anterior cingulate cortex—ACC) showed increased functional connectivity in controls when compared with stress-recovered participants. Results of increased functional connectivity of the DMN at rest after chronic stress exposure are consistent with those previously reported (Soares et al., 2013). More recently, Vaisvaser et al. (2013) evidenced similar results using an acute social stress model.

In the current study, we explored further the plasticity of the RSNs after recovery from the impact of chronic stress-induced changes and showed for the first time that all RSNs, with the exception of the DAN and AN, displayed a functional recovery after the cessation of the exposure to stress. Notably, the comparison with controls allowed us to observe a return to the initial levels the functional connectivity of the DMN, VAN, and AN, but still a sustained pattern of increased functional connectivity of the DAN, SMN, and VN networks.

These results suggest that DAN, SMN, and VN are less plastic when recovering from the impact of stress exposure. The DAN network has been associated with top-down attention processes as inhibitory control, working memory, and response selection. These cognitive processes depend upon the prefrontal integrity (dorsal frontal regions), which are brain regions vulnerable to the effects of stress (Cerqueira et al., 2007). Indeed, animal studies evidenced stress-related prefrontal remodeling (e.g., selective atrophy of the prefrontal cortex, elimination of dendritic spines) after chronic stress exposure (Cerqueira et al., 2007; Gourley et al., 2013). This stress-related prefrontal structural reorganization has been associated with impaired perceptual attention, behavioral flexibility, and decision making in rodents and humans (Cerqueira et al., 2005; Dias-Ferreira et al., 2009; Soares et al., 2012; Yuen et al., 2012). Interestingly, studies analysing the recovery of posttraumatic stress disorder reported that an increased thickness of the dorsolateral prefrontal cortex was associated with greater symptomatic alleviation (Lyoo et al., 2011). The concomitant SMN and VN sustained increased functional connectivity are possibly associated with a motor and visual readiness state that is required for the stress response. This specific pattern of plasticity suggests that some RSNs may be a tool for monitoring effective anti-stress interventions, similar to that proposed to verify the effect of the treatments in several neuropsychiatric diseases (Achard and Bullmore, 2007).

Besides the functional plastic recovery in the connectivity of the RSNs at rest, we also observed a continuum in the pattern of deactivation—that is, there was an increased deactivation from stress toward the control group in all the RSNs. DMN deactivation has been associated with reallocation of attentional resources to cognitively demanding tasks (Hu et al., 2013). Moreover, task-induced RSNs deactivation is correlated with behavioral performance: for example, stronger DMN deactivation in a working memory task predicts better performance (Uddin et al., 2009; Mayer et al., 2010). Increased deactivation observed in our control group and in our stress-recovered participants (comparing with stress). Additionally, abnormal patterns of RSNs deactivation have been associated with several neuropsychiatric diseases (Pomarol-Clotet et al., 2008; Guerrero-Pedraza et al., 2012).

This study shows that while the functional remodeling of RSNs endures, the structural changes (volumetry) of the brain areas involving these networks is still absent after this period of recovery, as no significant areas of expansion or constriction were found in the networks between stress and stress recovered participants; however, in contrast to the difference previously reported in the volumetry of the DMN after stress exposure (Soares et al., 2013) we also did not find significant differences between stress recovered and controls. Difference in results may be related with the limited sample size; the fact that our groups did not differ in physiological cortisol levels and finally, because no direct comparisons were made between stress and control groups.

In summary, the present study contributes to better understand the plastic phenomena that occur in RSNs after the cessation of stress exposure. While we have previously shown the existence of stress-related impairments in the activation-deactivation of RSNs (Soares et al., 2013), here we demonstrate that a functional remodeling of the activation-deactivation pattern of the RSNs takes place following chronic-stress recovery. Although promising, our results should be interpreted with caution mainly due to the reduced size of our sample; therefore, future studies should try to replicate these observations in a larger sample, ideally using exactly the same participants in all conditions, as controls, stressed, and after recovery.

## Author contributions

José M. Soares and Adriana Sampaio contributed in literature search, figures, study design, data collection, data analysis, data interpretation, and writing. Paulo Marques and Luís M. Ferreira as contributed in data collection and data analysis. Nadine C. Santos, Fernanda Marques, Joana A. Palha, João J. Cerqueira, and Nuno Sousa contributed in study design, data interpretation, and writing.

### Conflict of interest statement

The authors declare that the research was conducted in the absence of any commercial or financial relationships that could be construed as a potential conflict of interest.
